# Supplementary material for: Content-rich biological network constructed by mining PubMed abstracts
Source: BMC Bioinformatics. 2004 Oct 8;5:147. doi: 10.1186/1471-2105-5-147 (PMC528731; doi:10.1186/1471-2105-5-147)
Supplement: Additional File 2 — The original results of the above study (non-essential files are deleted to keep the file size under the limit set by BMC bioinformatics). [file 1471-2105-5-147-S2.bz2 › chilibotAdditionalFile2/dip50/290ID1833185E1120/html/CDC2_CCNB1.html]

 


 **CDC2** and **CCNB1** 
  
Found 825 abstracts in PubMed, retrieved 50.  
 

 What does Google say? 
 PDF only 
| .edu only 

---

**Interactive relationship** (e.g. stimulation, inhibition, etc)

**Inhibitory relationship**- The prolongation of G2 phase arrest after irradiation in the acidic pH environment appeared to be related at least in part to a prolongation of the phosphorylation of  **CDC2** , which inhibited cyclin B1  [ **CCNB1** ]   **CDC2**  kinase activity.  Ref: 12492371 Radiat Res, 2003
- Both down regulation of cell cycle regulators p34cdc2  [ **CDC2** ] , cyclin B1  [ **CCNB1** ]  and cdc25C and decreased association between p34cdc2  [ **CDC2** ]  and cyclin B1  [ **CCNB1** ]  were found to be associated with the activity of  **cdc2**  kinase abated after the H. pylori infection.  Ref: 12678330 Scand J Gastroenterol, 2003
- 3 1.5 mM caffeine that relieves cells from G 2 M arrest also inhibits As 2 O 3 induced Deltavarphi m collapse and apoptosis, 4 1.0 & ; mgr ; M As 2 O 3 increases the expression of both cyclin B  [ **CCNB1** ]  1 and hCDC20 whereas it inhibits Tyr15 phosphorylation of p34  **cdc2** .  Ref: 12835721 Leukemia< MedlineTA>Leukemia, 2003
- Thus, the impairment in cell cycle progression could not be explained by a lack of cyclin B1  [ **CCNB1** ] , or the ability of  **Cdc2**  and cyclin B1  [ **CCNB1** ]  to associate, but instead resulted, at least in part, from impaired  **Cdc2**  activity.  Ref: 12883482 Hepatology, 2003
- The ER clusters can be maintained by inhibiting the decrease in cdk1  [ **CDC2** ]  cyclin B  [ **CCNB1** ]  activity by using the proteasome inhibitor MG132, or by microinjecting excess cyclin B  [ **CCNB1** ] .  Ref: 12529444 Mol Biol Cell, 2003
- The addition of butyrolactone I, which is an inhibitor of CDK1  [ **CDC2** ]  and CDK2, to the p53 negative cells reduced the floating round cell population and induced the disappearance of cyclin B1  [ **CCNB1** ] .  Ref: 12646262 Arch Biochem Biophys, 2003
**Neutral relationship**- iii A dramatic slowing down in the rate of  **Cdc2**  activation is detected at concentrations of cyclin B  [ **CCNB1** ]  marginally above the activation threshold.  Ref: 12509509 Proc Natl Acad Sci U S A, 2003
- Immunoblot analysis revealed that ethanol metabolism had little affect on total  **Cdc2**  content in these cells, but resulted in the accumulation of up to 20 times the amount of cyclin B1  [ **CCNB1** ] , indicating that cyclin B1  [ **CCNB1** ]  was available for formation of  **Cdc2**  cyclin B1  [ **CCNB1** ]  complexes.  Ref: 12883482 Hepatology, 2003
- **Cdc2**  regulation also involves positive feedback, with active  **Cdc2**  cyclin B  [ **CCNB1** ]  stimulating its activator Cdc25 refs 5 7 and inactivating its inhibitors Wee1 and Myt1 refs 8 11.  Ref: 12629549 Nat Cell Biol, 2003
- **Cdc2**  is positively regulated by association with cyclin B1  [ **CCNB1** ] , and negatively regulated by phosphorylation of amino acids Thr14 and Tyr15.  Ref: 12883482 Hepatology, 2003
- Progression through mitosis occurs because cyclin B  [ **CCNB1** ]   **Cdc2**  activation induces the anaphase promoting complex APC to cause cyclin B  [ **CCNB1** ]  destruction and mitotic exit.  Ref: 12791267 Dev Cell, 2003
- These dynamic changes in cyclin B1  [ **CCNB1** ]   **Cdc2**  localization are regulated by phosphorylation of four serines within a region of cyclin B1  [ **CCNB1** ]  known as the cytoplasmic retention sequence CRS.  Ref: 12612056 Mol Cancer Res, 2003
- Consistent with these observations, CDK1  [ **CDC2** ]  activity was maintained at high levels, along with stable cyclin B1  [ **CCNB1** ] , in p53 negative cells.  Ref: 12646262 Arch Biochem Biophys, 2003

**Non-interactive relationship** (e.g. studied together, co-existance, homology, etc.)

- These results suggest that the RRASK motif in Xenopus cyclin B2 plays an important role in defining the substrate specificity of the cyclin B  [ **CCNB1** ]   **Cdc2**  complex.  Ref: 12754270 J Biol Chem, 2003
- The cyclin B1  [ **CCNB1** ]   **Cdc2**  complex regulates many of the dramatic cellular rearrangements observed at mitosis.  Ref: 12612056 Mol Cancer Res, 2003
- Western blot analysis revealed that oxLDL stimulated cell proliferation was associated with significant increases in the expression of proteins that regulate entry into and progression through the cell cycle cell division cycle 2  [ **CDC2** ] , cyclin dependent kinase cdk  [ **CDC2** ]  2, cdk  [ **CDC2** ]  4, cyclin B1  [ **CCNB1** ] , cyclin D1, and PCNA.  Ref: 12529257 Am J Physiol Heart Circ Physiol, 2003
- Finally, forced expression of the NF YA protein in cells committed to differentiate leads to an impairment in the down regulation of cyclin A, cyclin B1  [ **CCNB1** ] , and cdk1  [ **CDC2** ]  expression and is accompanied by a delay in myogenin expression.  Ref: 12857858 Mol Biol Cell, 2003
- Co immunoprecipitation revealed that 6 times more  **Cdc2**  cyclin B1  [ **CCNB1** ]  complexes were present in the ethanol treated cells compared with the controls.  Ref: 12883482 Hepatology, 2003
- Earlier we showed that U L 13 and ICP22 mediate the stabilization of  **cdc2**  and the replacement of its cellular partner, cyclin B  [ **CCNB1** ] , with the viral DNA polymerase processivity factor U L 42.  Ref: 12665617 Proc Natl Acad Sci U S A, 2003
- This was associated with earlier expression of the cell cycle promoting cyclin A2, cyclin E, cyclin B1  [ **CCNB1** ] , cyclin F and cyclin dependent kinase 1  [ **CDC2** ]  Cdk 1  [ **CDC2** ]  genes and diminished protein levels of Cdk  [ **CDC2** ]  inhibitor p21Cip1.  Ref: 12867420 J Biol ChemJ Biol Chem, 2003
- For example, in frog egg extracts, Cdk1  [ **CDC2** ]  cyclin B  [ **CCNB1** ]  catalyzes entry into mitosis but cannot trigger DNA replication.  Ref: 12738867 Science, 2003
- While G2 arrest is correlated with decreased cyclin B1  [ **CCNB1** ]   **CDC2**  kinase activity, the mechanisms associated with initiation and maintenance of the arrest are quite different.  Ref: 12492370 Radiat Res, 2003
- HEI10 is itself a specific in vitro substrate of purified cyclin B  [ **CCNB1** ]   **cdc2** , with a TPVR motif as primary phosphorylation site.  Ref: 12612082 Mol Cell Biol, 2003
- Cyclin B1  [ **CCNB1** ]  Cdk1  [ **CDC2** ]  is the key initiator of mitosis, but when and where activation occurs has not been precisely determined in mammalian cells.  Ref: 12524548 Nat Cell Biol, 2003
- Monensin decreased the levels of CDK2, CDK6,  **cdc2** , cyclin A, cyclin B1  [ **CCNB1** ] , cyclin D1 and cyclin E proteins but did not alter CDK4 protein.  Ref: 12792794 Int J Oncol, 2003
- cyclins accumulate and produce active mitotic  **Cdc2**  cyclin B  [ **CCNB1** ] .  Ref: 12629549 Nat Cell Biol, 2003
- In this study, we examined the effects of DIF 1 on the progesterone induced germinal vesicle breakdown in Xenopus laevis, which is thought to be mediated by a decrease in intracellular cAMP and the subsequent activation of mitogen activated protein kinase MAPK and maturation promoting factor, a complex of  **cdc2**  and cyclin B  [ **CCNB1** ] , which regulates germinal vesicle breakdown.  Ref: 12559368 Eur J Pharmacol, 2003
- Increased levels of cyclin B1  [ **CCNB1** ]  and enhanced activity of its associated cyclin dependent kinase  **cdc2**  became apparent during the clonal evolution of p210 bcr abl transduced 32D cell clones lacking p53.  Ref: 12801837 Haematologica, 2003
- In the mathematical model, the toggle switch is created by positive feedback in the phosphorylation reactions controlling the activity of  **Cdc2** , a protein kinase bound to its regulatory subunit, cyclin B  [ **CCNB1** ] .  Ref: 12509509 Proc Natl Acad Sci U S A, 2003
- A role for cdk1  [ **CDC2** ]  cyclin B  [ **CCNB1** ]  in ER organization is further suggested by the finding that the cdk  [ **CDC2** ]  inhibitor roscovitine causes the loss of ER clusters in MII eggs.  Ref: 12529444 Mol Biol Cell, 2003
- In order to understand the mechanism s of tumor promotion by MY and MG, we have studied the levels of PCNA, a marker of cell proliferation and cell cycle regulatory proteins, cyclin D1, and its associated kinase, cdk4, cyclin B1  [ **CCNB1** ] , and associated kinase,  **cdc2** .  Ref: 12616621 Teratog Carcinog Mutagen, 2003
- Positive staining for cyclin D1, cyclin E, cyclin A, cyclin B1  [ **CCNB1** ] , cdk2, cdk4,  **cdc2** , p53, p21, and p27 was observed in 63%, 66%, 31%, 32%, 51%, 77%, 71%, 43%, 35%, and 60% of the 82 carcinomas, respectively.  Ref: 12792921 Hum Pathol, 2003
- Moreover, cDNA array analysis showed that PC SPES caused up regulation of p21 WAF1 CIP1 and decreased expression of cyclin B  [ **CCNB1** ] , Nedd8,  **cdc2** , skp1, PCNA, MAD2L1, cyclin H, CKS2, E2F, Rbx1, MCM2, MCM5, Mpp2, Cullin Cul4A, Cks1p9 and McM7, which are involved in cell cycle progression.  Ref: 12695690 Cell Cycle< MedlineTA>Cell Cycle, 2003
- Activation of cyclin B  [ **CCNB1** ]   **Cdc2**  is an absolute requirement for entry into mitosis, but other protein kinase pathways that also have mitotic functions are activated during G 2 M progression.  Ref: 12609978 J Biol Chem, 2003
- The gene expression of cyclin B  [ **CCNB1** ]  and p34cdc2  [ **CDC2** ]  was increased after the 1 day treatment, reverted to the control level at 2 and 12 weeks of treatment, and was remarkably increased after the 52 week treatment.  Ref: 12841865 Cancer Sci, 2003
- Here, we show that Cdk1  [ **CDC2** ]  cyclin B1  [ **CCNB1** ]  has cryptic S phase promoting abilities that can be unmasked by relocating it from the cytoplasm to the nucleus and moderately stimulating its activity.  Ref: 12738867 Science, 2003
- p21 Waf1 Cip1 exerts a key role in driving this G 2 exit both by inhibiting cyclin B1  [ **CCNB1** ]  Cdk1  [ **CDC2** ]  and cyclin A Cdk1  [ **CDC2** ]  2 complexes, which control G 2 M progression, and by blocking the phosphorylation of pRb family proteins.  Ref: 12881433 EMBO JEMBO J, 2003
- Western and Northern blot hybridization showed an increased expression of both cyclin D1 and its associated kinase cdk4, and cyclin B1  [ **CCNB1** ]  and its associated kinase  **cdc2** , in livers of rats administered MY and MG after administration of DEN as compared to untreated or DEN controls.  Ref: 12616621 Teratog Carcinog Mutagen, 2003
- The mechanism of this uncoupling involves the cyclin B  [ **CCNB1** ]   **Cdc2**  dependent proteolytic cleavage of the N terminal ERK binding domain of MEK1 and the phosphorylation of Thr 286.  Ref: 12609978 J Biol Chem, 2003
- The interphase cytoplasmic location of cyclin B1  [ **CCNB1** ]   **Cdc2**  reflects continuous, albeit slow, nuclear import and much more rapid nuclear export.  Ref: 12612056 Mol Cancer Res, 2003
- Doxorubicin treatment did not alter cyclin B  [ **CCNB1** ]  or  **cdc2**  protein level, which is known as the essential component of G2 M transition.  Ref: 12579337 Int J Mol Med, 2003
- A stronger G2 M arrest by these combinations was accompanied by a substantial decrease in the levels of  **cdc2** , cyclin B1  [ **CCNB1** ]  and cdc25C.  Ref: 12866029 Int J CancerInt J Cancer, 2003
- These results suggest that G2 arrest induced by repeated TA treatment might be p53 dependent, via activation of cyclin G1, rather than inhibition of cyclin B1  [ **CCNB1** ]   **cdc2**  complex, and inhibitors holding S phase progression might be p27 and p57.  Ref: 12526100 Exp Mol Med, 2002
- 2 ME treatment results in phosphorylation of cdc25C without any significant alterations in the expression of cyclin B1  [ **CCNB1** ]  or p34cdc2  [ **CDC2** ] .  Ref: 12584169 Carcinogenesis, 2003
- We show that this conserved sequence in Xenopus cyclin B2, termed the RRASK motif, is required for the substrate recognition by the cyclin B  [ **CCNB1** ]   **Cdc2**  complex of Cdc25C.  Ref: 12754270 J Biol Chem, 2003
- These results suggest that the RRASK motif in Xenopus cyclin B2 plays an important role in defining the substrate specificity of the cyclin B  [ **CCNB1** ]   **Cdc2**  complex.  Ref: 12654907 J Biol Chem< MedlineTA>J Biol Chem, 2003
- The archival slides were incubated overnight at 4 degrees C using mouse anti human recombinant cyclin B1  [ **CCNB1** ]  polyclonal antibody or mouse anti Xenopus p34  **cdc2**  monoclonal antibody for immunohistochemistry Santa Cruz Biotech, Santa Cruz, CA.  Ref: 12821336 Gynecol Oncol, 2003
- When we examined the effects of this drug on ACHN cells, monensin decreased the levels of CDK2, CDK6,  **cdc2** , cyclin A and cyclin B1  [ **CCNB1** ]  proteins.  Ref: 12632079 Int J Oncol, 2003
- This genistein induced G2 M arrest in vitro was associated with a significant increase in the protein expression of phosphorylated p34  **cdc2**  and of cyclin B1  [ **CCNB1** ] .  Ref: 12668277 Cancer Lett, 2003
- Our results show that the mRNA and protein levels of the G 2 M regulators  **cdc2** , cyclin B1  [ **CCNB1** ]  and cdc25C are down regulated in MCF7TDX cells, while unaffected in MCF7 cells.  Ref: 12853888 Anticancer Drugs, 2003
- Flavopiridol treatment of docetaxel treated cells enhances the exit from mitosis with a more rapid decrease in mitotic markers including MPM 2 labeling and cyclin B1  [ **CCNB1** ]   **cdc2**  kinase activity.  Ref: 12813134 Mol Cancer Ther< MedlineTA>Mol Cancer Ther, 2003
- Degradation of Tome 1 during G1 allows for wee 1 accumulation during interphase, thereby providing a critical link between the APC and SCF pathways in regulation of cdk1  [ **CDC2** ]  cyclin B  [ **CCNB1** ]  activity and thus mitotic entry and exit.  Ref: 12679038 Cell, 2003
- Tome 1 is a cytosolic protein required for proper activation of cdk1  [ **CDC2** ]  cyclin B  [ **CCNB1** ]  and mitotic entry.  Ref: 12679038 Cell, 2003
- This block is abolished when HuCdc6 is expressed together with a constitutively active Cyclin B  [ **CCNB1** ]  CDK1  [ **CDC2** ]  complex or with Cdc25B or Cdc25C.  Ref: 12554670 EMBO J, 2003
- cyclin D1, cyclin B  [ **CCNB1** ] , and p34cdc2  [ **CDC2** ]  were examined by RT PCR.  Ref: 12841865 Cancer Sci, 2003
- Entry into mitosis requires the activation of cdk1  [ **CDC2** ]  cyclin B  [ **CCNB1** ] , while mitotic exit is achieved when the same kinase activity decreases, as cyclin B  [ **CCNB1** ]  is degraded.  Ref: 12679038 Cell, 2003
- These results demonstrate that cyclin B  [ **CCNB1** ]   **Cdc2**  activity regulates signaling through the MAPK pathway in mitosis.  Ref: 12609978 J Biol Chem, 2003
- Deregulated activity of the cyclin B1  [ **CCNB1** ]   **cdc2**  complex may be involved in the loss of temporal co ordination of mitotic events and further free the barrier to genomic instability of CML clonal myeloid progenitors lacking p53.  Ref: 12801837 Haematologica, 2003
- Furthermore, cyclin A CDK1  [ **CDC2** ]  2, cyclin B  [ **CCNB1** ]  CDK1  [ **CDC2** ]  2 and cyclin E CDK2 complexes can phosphorylate multiple p73 isoforms in vitro at threonine 86 T86.  Ref: 12676926 J Biol Chem< MedlineTA>J Biol Chem, 2003
- Collectively, these data demonstrate a new role for Erk in mitotic regulation, identify the Ser 101 directed kinase, and provide a picture of cyclin B1  [ **CCNB1** ]   **Cdc2**  regulation by the combinatorial action of distinct kinases.  Ref: 12612056 Mol Cancer Res, 2003
- We have previously found that the overexpression of p53 causes G 2 arrest and represses the synthesis of cyclin dependent kinase 1  [ **CDC2** ]  and cyclin B1  [ **CCNB1** ] , two proteins required for cells to traverse from G 2 into M.  Ref: 12874009 Cancer Res, 2003
- Two hypotheses can explain this observation Either Cdk1  [ **CDC2** ]  cyclin B  [ **CCNB1** ]  fails to recognize the key substrates of its S phase promoting counterparts, or its activity is somehow regulated to prevent it from activating DNA synthesis.  Ref: 12738867 Science, 2003
- We investigated the effects of an acidic environment on the G2 M phase arrest, apoptosis, clonogenic death, and changes in cyclin B1  [ **CCNB1** ]   **CDC2**  kinase activity caused by a 4 Gy irradiation in RKO.C human colorectal cancer cells in vitro.  Ref: 12492371 Radiat Res, 2003
- We confirm that the response of  **Cdc2**  to non degradable cyclin B  [ **CCNB1** ]  is temporally abrupt and switch like, as would be expected if  **Cdc2**  activation were bistable.  Ref: 12629549 Nat Cell Biol, 2003
- The increased sensitivity to etoposide induced G 2 M arrest was associated with disruption of cell cycle related events, including hyperphosphorylation of p34  **cdc2**  kinase, change in cdc25C expression and phosphorylation, and alteration in cyclin B1  [ **CCNB1** ]  expression.  Ref: 12569391 Br J Cancer, 2003
- Both immunoreactivities to cyclin B  [ **CCNB1** ]  and p34  **cdc2**  were strongly correlated with the HPV infection P = 0.0001 and the histological types P = 0.0001 between CIN and SCC, being strongly correlated with each other alpha 0.62954, P = 0.0001.  Ref: 12821336 Gynecol Oncol, 2003
- Cells exit mitosis in a specific time window without cytokinesis with a decrease in cyclin B1  [ **CCNB1** ]  cdc 2  [ **CDC2** ]  kinase activity and MPM 2 labeling.  Ref: 12813134 Mol Cancer Ther< MedlineTA>Mol Cancer Ther, 2003
- Genetic analysis of fission yeast and physiological studies of vertebrate and invertebrate oocytes showed that activation of cyclin B  [ **CCNB1** ]   **cdc2**  kinase triggers mitosis.  Ref: 12836412 Med Sci Paris , 2003
- We conclude that cyclin B1  [ **CCNB1** ]  Cdk1  [ **CDC2** ]  is first activated in the cytoplasm.  Ref: 12524548 Nat Cell Biol, 2003
- To ensure that cyclin B  [ **CCNB1** ]   **Cdc2**  does not prematurely activate the APC in early mitosis, there must be a mechanism delaying APC activation.  Ref: 12791267 Dev Cell, 2003
- The RRASK Motif in Xenopus Cyclin B2 Is Required for the Substrate Recognition of Cdc25C by the Cyclin B  [ **CCNB1** ]   **Cdc2**  Complex.  Ref: 12754270 J Biol Chem, 2003
- In contrast, pretreatment with flavopiridol prevents cells from entering mitosis by inhibiting cyclin B1  [ **CCNB1** ]  cdc 2  [ **CDC2** ]  kinase activity, thus antagonizing the docetaxel effect.  Ref: 12813134 Mol Cancer Ther< MedlineTA>Mol Cancer Ther, 2003
- The RRASK motif in Xenopus cyclin B2 is required for the substrate recognition of Cdc25C by the cyclin B  [ **CCNB1** ]   **Cdc2**  complex.  Ref: 12654907 J Biol Chem< MedlineTA>J Biol Chem, 2003
- Active cyclin B1  [ **CCNB1** ]  Cdk1  [ **CDC2** ]  first appears on centrosomes in prophase.  Ref: 12524548 Nat Cell Biol, 2003
- In the early embryonic cell cycle,  **Cdc2**  cyclin B  [ **CCNB1** ]  functions like an autonomous oscillator, whose robust biochemical rhythm continues even when DNA replication or mitosis is blocked.  Ref: 12629549 Nat Cell Biol, 2003
- Expression levels of cyclin B1  [ **CCNB1** ]  and  **cdc2**  were similar although the cyclin B1  [ **CCNB1** ]  level was generally low, decreased from day 1 to basal levels at day 3 and persisted at a low level till day 7.  Ref: 12526100 Exp Mol Med, 2002
- We show that this conserved sequence in Xenopus cyclin B2, termed the RRASK motif, is required for the substrate recognition by the cyclin B  [ **CCNB1** ]   **Cdc2**  complex of Cdc25C.  Ref: 12654907 J Biol Chem< MedlineTA>J Biol Chem, 2002
- The effect of STP on cell cycle arrest induced by X irradiation was studied in two cell lines by using flow cytometry, Western Blotting was performed to indicate the changes of cyclin B1  [ **CCNB1** ]  and  **cdc2**  protein levels.  Ref: 12579796 Yao Xue Xue Bao, 2002
